# Work Productivity Loss and Activity Impairment in Parents of Children With Juvenile Idiopathic Arthritis

**DOI:** 10.1002/acr2.90157

**Published:** 2026-09-24

**Authors:** Deborah A. Marshall, Rodrigo Dal Ben, Gillian R. Currie, Rae S. M. Yeung, Sebastiaan J. Vastert, Nico Wulffraat, Joost F. Swart, Susanne Benseler, the UCAN CAN‐DU and UCAN CURE Consortia, Rae Yeung, Shirley Tse, Deborah Levy, Ronald Laxer, Brian Feldman, Elizaveta Limenis, Ruud Verstegen, Lynn Spiegel, Rayfel Schneider, Evelyn Rozenblyum, Jennifer Ji Young Lee, Alisa Rachlis, Susanne Benseler, Nicole Johnson, Muhammed Dhalla, Paivi Miettunen, Heinrike Schmeling, Marinka Twilt, Rebeka Stevenson, Deborah Marshall, Andrea Human, David Cabral, Jaime Guzman, Kim Morishita, Kristin Houghton, Lori Tucker, Tommy Gerschman, Herman Tam, Mercedes Chan, Ross Petty, Roman Jurencak, Nadia Luca, Ciarán Duffy, Tala El Tal, Roberta Berard, Jonathan Park, Erkan Demirkaya, Lily Lim, Adam Huber, Bianca Lang, Elizabeth Stringer, Chelsea DeCoste, Suzanne Ramsey, Dax G. Rumsey, Daniah Basodan, Lillian Lim, Hon Yan Ng, Jeanine McColl, Tara McGrath, Michelle Batthish, Tania Cellucci, Liane Heale, Claire LeBlanc, Gaëlle Chédeville, Rosie Scuccimarri, Sarah Campillo, Piya Lahiry, Paul Dancey, Nickolas Blanchette, Alan Rosenberg, Mehul Jariwala, Tristan Kerr, Kate Neufeld, Clare Hutchinson, Gordon Soon, Dieneke Schonenberg, Mariken Gruppen, Merlijn van den Berg, Giske Biesbroek, Philomine van Pelt, Marleen Verkaaik, Sylvia Kamphuis, Danielle Brinkman, Petra Hissink Muller, Ellen Schatorje, Esther Hoppenreijs, Annet van Royen‐Kerkhof, Sebastiaan Vastert, Berent Prakken, Erika Van Nieuwenhove, Joost Swart, Marc Jansen, Nico Wulffraat, Elizabeth Legger, Wineke Armbrust, Joseph Cafazzo, Maarten IJzerman, Alexander Mosoiu, Amy Xu, Arthur Cheng, Bruno Pereira, Trang Duong, Harper Cheng, Brenleigh Jebb, Gillian R. Currie, Rodrigo Dal Ben, Ravneet Sran, Elodie Boudes, Michelle Kip, Sytze de Rook, Regina de Geus

**Affiliations:** ^1^ Department of Community Health Sciences, Cumming School of Medicine University of Calgary Calgary Alberta Canada; ^2^ Alberta Children's Hospital Research Institute University of Calgary Calgary Alberta Canada; ^3^ Department of Medicine, Cumming School of Medicine University of Calgary Calgary Alberta Canada; ^4^ Department of Pediatrics, Cumming School of Medicine University of Calgary Calgary Alberta Canada; ^5^ Department of Paediatrics, Immunology and Medical Science, The Hospital for Sick Children University of Toronto Toronto Ontario Canada; ^6^ Department of Pediatric Immunology and Rheumatology Wilhelmina Children's Hospital/UMC Utrecht Utrecht the Netherlands; ^7^ Faculty of Medicine University Medical Center Utrecht Utrecht the Netherlands; ^8^ Children's Health Ireland Dublin Ireland

## Abstract

**Objective:**

To determine the impact of juvenile idiopathic arthritis (JIA) on work productivity loss and daily activities of parents.

**Methods:**

The multicenter Canada‐Netherlands Understanding Childhood Arthritis Network (UCAN) CAN‐DU and CURE study captures consecutive clinical and patient‐reported data for patients and parents since 2019. Parents’ responses to the Work Productivity and Activity Impairment, Specific Health Problem related to their child's JIA were combined with demographic information and clinical reports from enrollment to 12 months. Demographic and clinical data were summarized, and generalized estimating equations (GEEs) were used to estimate absenteeism, presenteeism, work impairment, daily activities impairment, and wage loss. GEE covariates included JIA activity state, time from study enrollment, patient age group, and patient age at diagnosis.

**Results:**

Of the 754 parents, 635 were mothers with median age of 42 years (interquartile range = 10 years). Changes in work commitment were reported by 79 parents, primarily reducing working hours (n = 55). Over 12 months, because of their child's JIA, there was an estimated 6% of missed work time (absenteeism), 14% of working time impairment (presenteeism), 12% of combined work impairment, and 16% of daily activities impairment. Absenteeism resulted in an estimated average annual wage loss of €3,510 per family. Work impairment and activity impairment estimates were higher for parents of children with more severe disease activity.

**Conclusion:**

This study is the first to capture the adjusted work impairment and activity impairment for a large international cohort of parents of children across all JIA subtypes and at different stages of treatment.

## INTRODUCTION

Juvenile idiopathic arthritis (JIA) is the most common rheumatic disease in children, frequently resulting in severe joint pain and impaired mobility and often carrying lifelong disability risks.^1^, ^2^ JIA also impacts parent's employment and overall quality of life, and results in substantial health care costs and personal costs for families.^3^, ^4^ The significant economic impact of JIA has been highlighted in three systematic reviews.^5^, ^6^, ^7^ Direct health care costs are largely driven by treatment with biologics and health care appointments, whereas costs outside the health care system are mostly based on productivity loss among parents.

**SIGNIFICANCE & INNOVATIONS**

- This is the first study to fully investigate the impact of juvenile idiopathic arthritis (JIA) on parent employment and usual activities using a standardized measure of work impairment and activity impairment in a large international cohort (n = 754).
- This study contributes to our understanding of the socioeconomic impact of JIA, specifically on parents’ work impairment.
- The findings can support changes in practice or policy to support families caring for children with JIA facing employment‐related challenges.
- Future research could further examine the determinants of larger work impairment and activity impairment to help identify parents at risk of employment‐related challenges.

A scoping review of cost and economic evaluations in JIA studies identified that work productivity is not frequently included in these studies.^8^ When included, work impacts are measured differently across studies, with very few using standardized instruments making it very challenging to make comparisons. Furthermore, when work impacts are included, most studies focus on time missed from work. However, time missed from work is just one component of work productivity loss. A more complete picture of the impact of JIA on parent's economic activities would also include metrics such as labor force participation, presenteeism, and effects on usual activities. One study investigated these metrics with 61 parents of children from nine tertiary centers across the United States, France, Germany, the Netherlands, and United Kingdom with one specific subtype of JIA—systemic JIA.^9^ Shenoi et al^9^ reported that approximately one‐third of parents reduced their working hours or stopped working altogether due to their child's diagnosis. Overall, using a standardized instrument, the authors estimated a 15% work impairment and 17% impairment on daily activities. Using the same standardized instrument, other studies reported on work and activity impairments in inflammatory bowel disease (IBD), a childhood illness similar to JIA in its inflammatory nature and biologic treatments.^10^, ^11^, ^12^, ^13^ A prospective international study^10^ observed families caring for children with IBD for a year and estimated a 51% work impairment for parents, with an annual wage loss estimated at €6,272 per family.

In summary, there remains limited evidence on the work impacts for parents of children with JIA. Work effects are not frequently included, are not measured in a standardized way, and tend to focus on missed work only. This study aimed to investigate work force participation, work impairment (including absenteeism and presenteeism), and activity impairment in parents of children with JIA. It estimates these outcomes over a one‐year period and includes the role of clinical features such as disease activity, disease duration, age at diagnosis, and patient age on these outcomes.

## MATERIALS AND METHODS

### Participants and study design

Parents were recruited as part of the Canada‐Netherlands Personalized Medicine Network in Childhood Arthritis and Rheumatic Diseases (UCAN CAN‐DU) and UCAN CURE: Precision decisions for childhood arthritis (see Appendix A for a list of UCAN CAN‐DU and UCAN CURE Consortia members). This was a prospective international study that observed 0‐ to 18‐year‐olds that were recently diagnosed with JIA, starting biologic treatment, or stopping biologic treatment. All pediatric rheumatology clinics from Canada and the Netherlands (n = 18) were part of the study.

Parents provided informed consent during their child's first clinical visit and completed baseline questionnaires. Follow‐up questionnaires were shared with families every three months until the end of their participation. All families were observed for at least 12 months, the period included in the present study. Data collected between July 8, 2019, and March 7, 2025, from parents that answered at least one Work Productivity and Activity Impairment, Specific Health Problem (WPAI‐SHP) questionnaire and that had complete data for patient age, JIA activity state, and patient age at diagnosis at each observation (covariates on statistical models, see the “Statistical analyses” section) were included. Ethical approval for this study was granted by the University of Calgary (Conjoint Health Research Ethics Board, REB17–1563), University of Toronto (CTO #1771), and University of Utrecht Ethical Board (18–474).

### Outcome measures

Main outcome measures come from the standardized questionnaire WPAI‐SHP.^14^ This instrument includes measures of absenteeism, presenteeism, work impairment, and activity impairment and has successfully captured productivity loss and activity impairment of rheumatoid arthritis patients^15^ and parents of children with systemic JIA.^9^ The WPAI‐SHP asks parents (1) whether they are employed; and employed respondents are asked to estimate, in the previous 7 days: (2) how many hours they worked, (3) how many hours they missed due to their child's disease, (4) how many hours they missed due to other reasons (eg, vacation), (5) to which extent their child's disease impacted their work productivity (discrete visual analog scale from 0 to 10), and (6) to which extent their child's disease impacted their daily activities (discrete visual analog scale from 0 to 10). From these six questions, the percentage of time missed from work (ie, absenteeism), impairment while working (ie, presenteeism), work impairment, and daily activities impairment are calculated.

We also estimated wage loss due to absenteeism by combining estimated absenteeism, hours worked, employment rate (as collected by WPAI‐SHP), and the 2024 Dutch hourly productivity loss cost^16^ (€39.88) in the following formula^10^:

$$\frac{\text{Absenteeism}}{100}\times 13\;\text{weeks}\times \text{Hours worked}\times \text{€}39.88\times \frac{\text{Employment rate}}{100}\times 1$$

The formula extrapolates the 7‐day recall period from WPAI‐SHP to 3‐month periods (see the “Data collection” section), which allows us to estimate wage loss from 3 months before enrollment until enrollment, from enrollment to 3 months and so on until 12 months of participation. The Dutch hourly productivity loss cost was selected because it is a recent standardized reference value. Importantly, wage loss estimates incorporated observed employment rate and weekly hours worked from WPAI‐SHP responses. Therefore, differences in employment participation and hours worked are already reflected in the estimated absenteeism‐related wage loss. Finally, as wage loss estimates are linear, the reader may rescale them to the preferred standardized value in their jurisdiction by multiplying our reported wage loss by an alternative hourly value and dividing the result by €39.88.

All estimates (ie, employment rate, hours worked, absenteeism, presenteeism, work impairment, activity impairment, and wage loss) were analyzed over time (baseline, 3, 6, 9, and 12 months) and in combination with clinically meaningful covariates (ie, patient age at diagnosis, patient age group, and JIA activity state) depending on model specifications (see the “Statistical analyses” section). Patients were categorized into four age groups (ie, 0–4, 5–9, 10–15, and 16+ years), and JIA activity state (ie, high, moderate, minimum, or inactive) for each patient was categorized based on the combination of JIA subtype and Juvenile Arthritis Disease Activity Score 10 (cJADAS10),^17^ collected during clinical visits. We used cJADAS10 scores that were closest in time to each WPAI‐SHP observation (median = 12 days apart, interquartile range [IQR] = 91). Missing cJADAS10 scores (26%) were linearly predicted using global physician assessment scores, which were strongly correlated with cJADAS10 (Pearson r = 0.86, *P* < 0.001). JIA activity state cutoffs were available for patients with oligoarthritis and polyarthritis. Cutoffs from polyarthritis were used for patients with any JIA subtypes other than oligoarthritis^17^ (eg, systemic JIA and enthesis‐related arthritis). cJADAS10 cutoffs by Trincianti et al^17^ are as follows: (1) oligoarthritis: (a) inactive disease ≤1.1, (b) minimal disease activity 1.2 to 4, (c) moderate disease activity 4.1–12, and (d) high disease activity >12; (2) polyarthritis: (a) inactive disease ≤2.5, (b) minimal disease activity 2.6 to 5, (c) moderate disease activity 5.1 to 16, and (d) high disease activity >16.

Finally, we also explored parent's demographic information (eg, changes in work commitment and age), and child's demographic and clinical information (eg, time of diagnosis, disease status, and JIA subtype classification)—as collected by demographic questionnaires and electronic case reports from clinical visits.

### Data collection

Data were captured prospectively in the UCAN e‐health platform, a multilingual web‐based application integrating clinical history and phenotype, medications, the multinomic biologic phenotype, and standardized and be‐spoke instruments to measure patient and parent‐reported outcomes. At enrollment, patients and parents were registered on the UCAN platform. Once enrolled, and every three months from enrollment, parents were invited by email to join a secure environment and complete questionnaires. From the invitation date, there was a 30‐day opportunity window to answer questionnaires (±14 days around each 3‐month mark). Following completion or the end of the opportunity window, questionnaires became unavailable until the next response window, three months later, when a new invitation was sent. All data, regardless of collection site, were managed by the Sick Kids Hospital, Toronto University. Data are available on reasonable request and according to the UCAN CAN‐DU and UCAN CURE data‐sharing policy.

### Statistical analyses

Descriptive analyses of parents’ demographic information and patients’ demographic and clinical information included counting cases and summarizing continuous data using mean and SD or median and IQR, when normality was not met. Normality was tested with Jarque‐Bera normality tests^18^ with critical values estimated via Monte Carlo simulations and significance values set at 0.05. Changes in work commitment, as reported by parents at enrollment, were described as proportions.

Using WPAI‐SHP official scoring,^14^ we calculated the percentage of time missed from work (ie, absenteeism), impairment while working (ie, presenteeism), work impairment, daily activities impairment, employment rate, and hours worked. We also estimated wage loss using the formula described in the “Outcome measures” section. Building on recent work with WPAI‐SHP,^10^ these scores were entered as outcome measures in generalized estimating equations (GEEs) that produced population‐averaged estimates, including robust confidence intervals,^19^ adjusting for time from enrollment, and clinically meaningful covariates: JIA activity state, patient age group, and patient age at diagnosis (refer to Supplementary Table S1 for an evaluation of the statistical role of each covariate). Our main analytical interest is on population‐averaged estimates rather than statistical significance. Unadjusted GEEs, that accounted for JIA activity state only, were also used. Based on our study longitudinal design and data completeness (missing outcomes are assumed to be missing at random), GEEs were used because it does not require normal distribution of outcomes, handle repeated measures of clustered data,^20^ and allow specification of correlation structure over time. To specify GEEs’ correlation structure, we compared independent (no correlation between observations), exchangeable (equal correlations over time), autoregressive (correlation declines exponentially over time), and unstructured (distinct correlation for each pair of observations) structures when modeling absenteeism with an adjusted model. Using the lowest likelihood under independence criterion,^21^ the autoregressive correlation structure yielded the best fit to the data. We used this correlation structure for all GEEs, adjusted or unadjusted, and outcome measures. Finally, GEEs’ goodness of fit was assessed with marginal *R* ^2^, that compares fitted with observed scores, returning a value from 0 (poor fit) to 1 (perfect fit).^22^ Please note that *R* ^2^_marginal_ values are not directly comparable with *R* ^2^ values from ordinary least squares models. Importantly, the covariates on our models were not selected to optimize fit, but to provide meaningful estimates of population‐averaged effects with practical significance in the context of our complex dataset.

## RESULTS

Overall, data from 754 families of children with JIA from 18 sites across the Netherlands (n = 410) and Canada (n = 344) were included. Out of five opportunities over the year to answer WPAI‐SHP questionnaires, completion ranged from one to five (61%, 64%, 56%, 50%, 43% of participants completing the questionnaire at each time point, respectively), an attrition rate in line with reports from other longitudinal studies.^23^

Table 1 displays demographics for responding parents and patients at enrollment. Most responding parents were female (84%), with a median age of 42 years (IQR = 10), with higher education degrees (64%), and living with spouse (65%). Most patients were female (62%) with a median age of 11 years (IQR = 9). The most prevalent JIA type was oligoarticular JIA with 44% of cases. The most common JIA activity state was moderate (46%). It is important to note missingness levels reported in Table 1 (eg, parent sex and education) regards only demographic reporting at enrollment and do not affect GEE estimates, which were based on WPAI‐SHP answers and complete observations of covariates at each time point.

**Table 1:** Characteristics of responding parents and children with JIA*

Variable	Overall	Canada	The Netherlands
Number of respondents	754	344	410
Parent sex, n (%)			
Female	635 (84.2)	289 (84.0)	346 (84.4)
Male	108 (14.3)	46 (13.4)	62 (15.1)
Missing	11 (1.5)	9 (2.6)	2 (0.5)
Parent age, median (IQR), y	42.0 (36.0–46.0)	41.0 (36.0–46.0)	42.0 (37.0–47.0)
Parent highest education level, n (%)			
Technical trade school or University	481 (63.8)	197 (57.3)	284 (69.3)
Grade school	65 (8.6)	34 (9.9)	31 (7.6)
Missing	208 (27.6)	113 (32.8)	95 (23.2)
Parent living with spouse, n (%)			
Yes	488 (64.7)	204 (59.3)	284 (69.3)
No	61 (8.1)	28 (8.1)	33 (8.0)
Missing	205 (27.2)	112 (32.6)	93 (22.7)
Biologic sex of patient with JIA, n (%)			
Female	467 (61.9)	213 (61.9)	254 (62.0)
Male	277 (36.7)	122 (35.5)	155 (37.8)
Missing	10 (1.3)	9 (2.6)	1 (0.2)
Age of patient with JIA, median (IQR)	11.0 (6.0–15.0)	11.0 (6.0–15.0)	12.0 (6.0–15.0)
JIA classification, n (%)			
Enthesitis‐related arthritis	86 (11.4)	46 (13.4)	40 (9.8)
Oligoarticular JIA^a^	330 (43.8)	131 (38.1)	199 (48.5)
Polyarticular RF‐negative JIA	181 (24.0)	77 (22.4)	104 (25.4)
Polyarticular RF‐positive JIA	34 (4.5)	14 (4.1)	20 (4.9)
Psoriatic arthritis	31 (4.1)	22 (6.4)	9 (2.2)
Systemic JIA	62 (8.2)	35 (10.2)	27 (6.6)
Undifferentiated JIA	30 (4.0)	19 (5.5)	11 (2.7)
Number of active joints, median (IQR)	2.0 (1.0–4.0)	2.0 (1.0–4.0)	2.0 (1.0–3.0)
JIA activity state, n (%)			
High	98 (13.0)	40 (11.6)	58 (14.1)
Moderate	347 (46.0)	168 (48.8)	179 (43.7)
Minimal	148 (19.6)	66 (19.2)	82 (20.0)
Inactive	161 (21.4)	70 (20.3)	91 (22.2)
Disease duration, median (IQR), mo	12.0 (5.0–39.0)	12.0 (5.0–39.0)	11.5 (4.0–39.2)

* Numbers are No. (%) unless otherwise noted. Missing data were ignored when calculating median (IQR). IQR, interquartile range; JIA, juvenile idiopathic arthritis; RF, rheumatoid factor.

^a^ Oligoarticular (if <6 months), n = 159; persistent oligoarticular JIA, n = 125; and extended oligoarticular JIA, n = 46.

### Employment status

Over the study period and JIA activity states, employment rate as measured by WPAI‐SHP was at 81%, with respondent parents working on average 28 hours per week—see Supplementary Table S2 for a breakdown of employment rate and work hours per week over time and JIA activity state. Nonetheless, there was an estimated annual difference of 8% in employment rates between parents caring for children with high JIA activity states (77%) and those caring for children with inactive JIA activity state (85%). Most responding parents and their partners or spouses had not reported changing their work commitment due to their child's JIA in the year before enrollment (n = 358, n = 381, respectively). For those that reported changes in work commitment (n = 79, n = 25, respectively), reducing working hours was the most common change (n = 55, n = 18, respectively) (Table 2). Less than five responding parents and spouses, all female, reported changing to more demanding job or increasing working hours.

**Table 2:** Changes in work commitment of parents of children with JIA*

Variable	Respondent	Partner or spouse
Number of respondents	746	746
Have you changed your work commitment because of your child's JIA, n (%)		
No change in work commitments	358 (48.0)	381 (51.1)
Yes	79 (10.6)	25 (3.4)
Missing	309 (41.4)	340 (45.6)
For those that answered yes to the previous question: how have you changed your work commitment because of your child's JIA, n (%)		
Reduced working hours	55 (69.6)	18 (72.0)
Stopped working altogether or changed to less demanding job	20 (25.1)	6 (24.0)
Other	<5	<5

* Numbers are No. (%). Change in work commitment was defined as any parent‐reported change in employment status, working hours, or job demands owing to the child's JIA, including increases and reductions in work commitment. Most reported changes reflected reduced work commitment. JIA, juvenile idiopathic arthritis.

### Absenteeism, presenteeism, work impairment, and activity impairment

**Figure 1:**
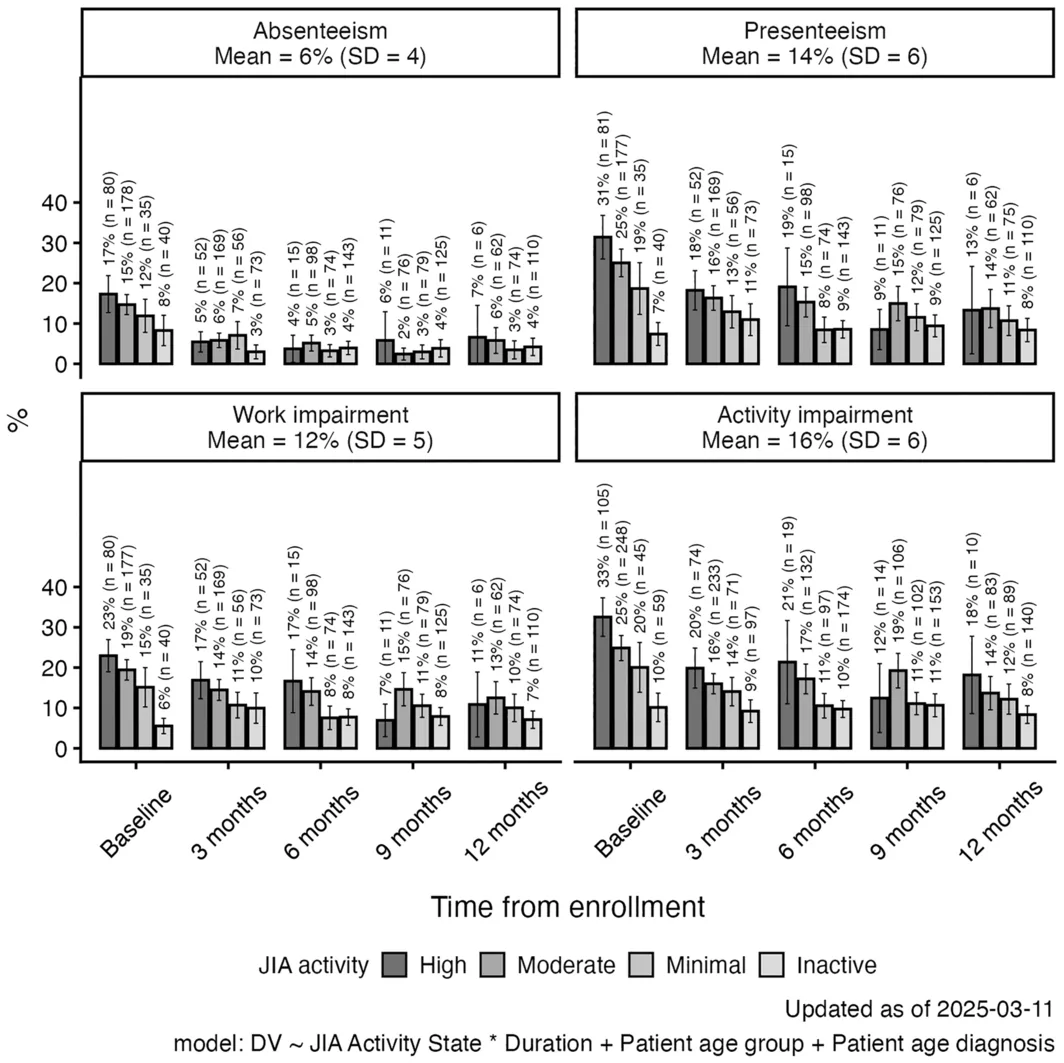
Responding parents’ estimated absenteeism, presenteeism, work impairment, and activity impairment over time and JIA activity state. Bars display adjusted means with robust 95% confidence intervals. Sample sizes are depicted in parenthesis for each outcome, time point, and JIA activity state. The varying numbers reflect the varying response rates over time. Adjusted means were estimated with generalized estimating equations having JIA activity state, time from enrollment, age group, and patient age at diagnosis as covariates. See Supplementary Table S2 for additional details. DV, dependent variables; JIA, juvenile idiopathic arthritis.

Figure 1 depicts estimates of absenteeism, presenteeism, work impairment, and activity impairment when adjusting for JIA activity state, time from enrollment, patient age group, JIA and patient age at diagnosis (*R* ^2^_marginal_ range = 0.11–0.13). All measures peaked at baseline and decreased over time thereafter. Overall, higher levels on all outcomes were observed for parents of patients with high JIA activity state in comparison to those caring for patients with moderate, minimum, or inactive JIA activity states. Over JIA activity states, parents experienced an annual average of 6% (SD = 4%) of absenteeism, 14% (SD = 6%) of presenteeism, 12% (SD = 5%) of work impairment, and 16% (SD = 6%) of activity impairment due to their child's JIA. The contrast between parents caring for children with high JIA activity state and those caring for children with inactive JIA activity state reveals an average annual difference of 7% in work impairment estimates (15% vs 8%, respectively) and 11% in activity impairment estimates (21% vs 10%, respectively).

### Wage loss

We estimate that, regardless of JIA activity state, parents experienced higher wage losses just before joining the study, a period in which half of the families received a formal diagnosis of JIA for their children (51%, n = 384). From 3 months before joining the study until 12 months of participation, we estimate that reporting parents lost an average of €3,510. Parents caring for children with high JIA activity state had an annual loss of €4,206 (SD = €586) whereas parents caring for children with inactive JIA activity state lost €2,773 (SD = €204) (Supplementary Figure S1; Supplementary Table S2). Importantly, employment rate and hours worked were comparable across countries (Supplementary Table S3).

## DISCUSSION

The study demonstrated substantial work impairment and daily activities impairments for parents of patients with JIA. About 10% of parents reported changing their work schedule in the previous year to accommodate caring for their JIA child, mostly via reducing their working hours. We estimated an annual 12% work impairment and 16% activity impairment. In addition, an average annual wage loss due to absenteeism of €3,510 was estimated across JIA activity states, with higher wage losses for parents of patients with high and moderate JIA. The highest impact of JIA on employment was found at study enrollment and decreased over time.

Despite the considerable socioeconomic impact of caring for patients with JIA, the published evidence on the topic is still scarce. This is one of the first studies to use a validated instrument to estimate work impairment and activity impairment of JIA parents. In contrast to Shenoi et al,^9^ we have a larger sample (n = 754 vs n = 61), diverse JIA types (eg, all subtypes compared to systematic JIA only), and diverse disease trajectory (ie, newly diagnosed and starting or stopping biologics compared to patients taking biologics). Parents were recruited across multiple jurisdictions, and our longitudinal design allowed us to investigate changes in productivity loss and activity impairment across time, all of which added to the generalizability of our findings. Importantly, wage loss estimates were not stratified by potentially relevant demographic and occupational variables such as parent sex, age, education, occupation, or industry. Future studies, according to its objectives, could provide more tailored sensitivity analyses of monetary productivity losses.

Our absenteeism estimate at enrollment is comparable to Shenoi et al^9^ (average of 13% vs 10%), and our presenteeism estimation is slightly higher (21% vs 11%). Nonetheless, the overall impact of JIA on work impairment and activity impairment decreases as treatment unfolds. It could be that families both adapt to life with arthritis and that prognosis improves following initial months of treatment. Overall, we estimate annual cost of absenteeism of €3,510, which is about four times higher than previously estimated,^24^ although previous studies have used different methods and populations. Importantly, JIA activity state was a key factor on work impairment and activity impairment, with parents of children with high and moderate JIA carrying a heavier burden than parents of children with minimal and inactive JIA. Compared with pediatric IBD, our estimates point to a lower parental work impairment, activity impairment, and annual costs. Previous studies in pediatric IBD reported productivity losses ranging from 21% to 32%, activity impairment of 25%, and annual absenteeism costs of €6,272.^10^, ^25^ Whereas IBD is a useful comparator given its similar chronicity, immune‐mediated pathophysiology, and long‐term treatment requirements, disease‐specific differences in clinical course and health care utilization could lead to differential work impact. Diagnostic procedures, biologic infusions, hospitalizations, and greater school absenteeism, may contribute to the higher productivity losses reported in pediatric IBD. Nevertheless, there is a substantial impact on parents of children with JIA.

In addition to the questions from WPAI‐SHP, we asked at baseline if both the responding parent and their spouse had previously had to make changes to their work commitment in the last year (eg, work less or more or switch to more or less challenging jobs). We found that about half of parents had not made changes to their work commitment. Of those that did, over 85% either reduced work hours, stopped working altogether, or changed to a less demanding job (Table 2). This compares to Shenoi et al^9^ who found that from 61 parents of children with sJIA, 64% had not changed work commitments, and of those that had 36% had reduced working hours or stopped working. Differences between our findings and those of Shenoi et al^9^ might be related to the timing of the questions and patients’ heterogeneity. We asked about any work changes that happened in the previous year to study enrollment, whereas Shenoi et al^9^ asked for changes since sJIA diagnosis. Also, we had diverse JIA subtypes and Shenoi et al^9^ focused on patients with sJIA. Other reviews of the impact of caring for a child with chronic illness have also found that parents are less likely to be participating in the labor force^26^, ^27^, ^28^ with one exception where there are short‐term reductions on participation and earnings but longer‐term increases.^29^ Our study only asked about work commitment changes at baseline, and future research should examine the trajectory of employment for parents of children with JIA, including the differential impacts on parents and spouses, and explore the dynamics of choices over time particularly for parents of children needing more support and who have to increase income to offset the cost of hiring help or purchasing equipment.

The study confirms the substantial socioeconomic impact of JIA on parents’ work productivity and daily activities. It also suggests that such impact will vary according to JIA severity and treatment course. This more complete picture can inform decision‐making at both policy and treatment level to improve the lives of families facing JIA.

## AUTHOR CONTRIBUTIONS

All authors contributed to at least one of the following manuscript preparation roles: conceptualization AND/OR methodology, software, investigation, formal analysis, data curation, visualization, and validation AND drafting or reviewing/editing the final draft. As corresponding author, Dr Currie confirms that all authors have provided the final approval of the version to be published and takes responsibility for the affirmations regarding article submission (eg, not under consideration by another journal), the integrity of the data presented, and the statements regarding compliance with institutional review board/Declaration of Helsinki requirements.

## Supporting information


**Disclosure form**.

**Table S1:** P‐values for each covariate and outcome measures (i.e., employment rate, absenteeism, presenteeism, work impairment, activity impairment) while keeping all remaining covariates constant (i.e., JIA activity state, time from enrolment, patient age group, patient age at diagnosis)
**Table S2:** Responding parents’ employment rate, hours worked, absenteeism, presenteeism, work impairment, activity impairment, and costs related to absenteeism, represented by child's JIA duration and JIA activity
**Table S3:** Responding parents’ employment rate and hours worked by country
**Figure S1:** Estimated wage loss due to absenteeism over time and by JIA activity state. Wage losses were estimated from 3 months prior to enrolment up to 12 months of participation.

## APPENDIX A UCAN CAN‐DU AND UCAN CURE CONSORTIA MEMBERS

Members of the UCAN CAN‐DU and UCAN CURE Consortia are as follows: The Hospital for Sick Children: Rae Yeung, Shirley Tse, Deborah Levy, Ronald Laxer, Brian Feldman, Elizaveta Limenis, Ruud Verstegen, Lynn Spiegel, Rayfel Schneider, Evelyn Rozenblyum, Jennifer Ji Young Lee, and Alisa Rachlis; University of Calgary: Susanne Benseler, Nicole Johnson, Muhammed Dhalla, Paivi Miettunen, Heinrike Schmeling, Marinka Twilt, Rebeka Stevenson, and Deborah Marshall; BC Children's Hospital: Andrea Human, David Cabral, Jaime Guzman, Kim Morishita, Kristin Houghton, Lori Tucker, Tommy Gerschman, Herman Tam, Mercedes Chan, and Ross Petty; Children's Hospital of Eastern Ontario: Roman Jurencak, Nadia Luca, Ciarán Duffy, and Tala El Tal; London Health Sciences Centre: Roberta Berard, Jonathan Park, and Erkan Demirkaya; Children's Hospital Health Centre Winnipeg: Lily Lim; IWK Health Centre: Adam Huber, Bianca Lang, Elizabeth Stringer, Chelsea DeCoste, and Suzanne Ramsey; Stollery Children's Hospital: Dax G. Rumsey, Daniah Basodan, Lillian Lim, Hon Yan Ng, Jeanine McColl, and Tara McGrath; McMaster Children's Hospital: Michelle Batthish, Tania Cellucci, and Liane Heale; Montreal Children's Hospital: Claire LeBlanc, Gaëlle Chédeville, Rosie Scuccimarri, Sarah Campillo, and Piya Lahiry; Janeway Children's/Memorial University: Paul Dancey; Trillium Health Partners: Nickolas Blanchette; University of Saskatchewan: Alan Rosenberg, Mehul Jariwala, Tristan Kerr, and Kate Neufeld; North York General Hospital: Clare Hutchinson and Gordon Soon; Emma Children's Hospital, Amsterdam UMC: Dieneke Schonenberg, Mariken Gruppen, Merlijn van den Berg, and Giske Biesbroek; Sophia Children's Hospital, EMC: Philomine van Pelt, Marleen Verkaaik, and Sylvia Kamphuis; Willem‐Alexander Children's Hospital, LUMC: Danielle Brinkman and Petra Hissink Muller; Amalia Children's Hospital, Radboudumc and Sint Maartenskinderkliniek: Ellen Schatorje and Esther Hoppenreijs; Wilhelmina Children's Hospital, UMCU: Annet van Royen‐Kerkhof, Sebastiaan Vastert, Berent Prakken, Erika Van Nieuwenhove, Joost Swart, Marc Jansen, and Nico Wulffraat; Beatrix Children's Hospital, UMCG: Elizabeth Legger and Wineke Armbrust; Centre for Global e‐health Innovation, University Health Network, University of Toronto: Joseph Cafazzo; Erasmus University and University of Melbourne: Maarten IJzerman; The Hospital for Sick Children: Alexander Mosoiu, Amy Xu, Arthur Cheng, Bruno Pereira, Trang Duong, Harper Cheng, and Brenleigh Jebb; University of Calgary: Gillian R. Currie, Rodrigo Dal Ben, Ravneet Sran, and Elodie Boudes; University of Twente: Michelle Kip; and University Medical Center Utrecht: Sytze de Rook and Regina de Geus.
