# Supplementary material for: Work Productivity Loss and Activity Impairment in Parents of Children With Juvenile Idiopathic Arthritis
Source: ACR Open Rheumatol. 2026 Sep 24;8(10):e90157. doi: 10.1002/acr2.90157 (PMC13612856; doi:10.1002/acr2.90157)
Supplement: Supplementary file 2 [file ACR2-8-e90157-s002.docx]

**Supplementary material**

| **Table S1. P-values for each covariate and outcome measures (i.e., employment rate, absenteeism, presenteeism, work impairment, activity impairment) while keeping all remaining covariates constant (i.e., JIA activity state, time from enrolment, patient age group, patient age at diagnosis)** | | | | | |
| --- | --- | --- | --- | --- | --- |
| Covariate | Employment rate | Absenteeism | Presenteeism | Work impairment | Activity impairment |
| JIA activity state | 0.8 | < .001 | < .001 | < .001 | < .001 |
| Time from enrolment | 0.75 | < .001 | < .001 | < .001 | < .001 |
| Patient age at diagnosis | 0.93 | 0.53 | 0.41 | 0.56 | 0.45 |
| Patient age group | 0.95 | 0.67 | 0.83 | 0.9 | 0.13 |
| JIA activity state x Time from enrolment (interaction term) | 0.36 | < .001 | < .001 | < .001 | < .001 |
| JIA activity state x Age group (interaction term) | 0.9 | 0.69 | 0.85 | 0.63 | 0.35 |
| P-values below the alpha level 0.05 indicate that a given covariate (i.e., JIA activity state, time from enrolment, patient age group, patient age at diagnosis), or interaction between covariates (i.e., JIA activity state x age group; JIA activity state x time from enrolment), detects statistically significant differences on a given outcome measure (i.e., employment rate, absenteeism, presenteeism, work impairment, activity impairment). Significant p-values indicate that included covariates are statistically relevant, on top of their a-priori theoretical and clinical relevance for JIA. All GEE models had the following specification: Outcome ~ JIA activity state * Time from enrolment + Patient age at diagnosis + JIA activity state * Patient age group.  GEE *R*^2^_marginal_ varied from: 0.01 to 0.13 | | | | | |

| **Table S2. Responding parents’ employment rate, hours worked, absenteeism, presenteeism, work impairment, activity impairment, and costs related to absenteeism, represented by child’s JIA duration and JIA activity** | | | | | |
| --- | --- | --- | --- | --- | --- |
| Duration | Dimension | High | Moderate | Minimal | Inactive |
| Baseline | Employment rate | 81 (75-87) | 79 (74-83) | 80 (70-90) | 81 (74-88) |
|  | Hours worked | 26 (23-29) | 26 (24-28) | 24 (19-29) | 26 (21-30) |
|  | Absenteeism | 17 (13-22) | 15 (12-17) | 12 (8-16) | 8 (5-12) |
|  | Presenteeism | 31 (26-37) | 25 (22-28) | 19 (12-25) | 7 (5-10) |
|  | Work impairment | 23 (19-27) | 19 (17-22) | 15 (10-20) | 6 (4-7) |
|  | Activity impairment | 33 (28-37) | 25 (22-28) | 20 (14-26) | 10 (7-14) |
|  | Wage loss | €1876 (€1131-€2842) | €1569 (€1139-€2081) | €1196 (€549-€2170) | €902 (€369-€1681) |
| 3 months | Employment rate | 81 (75-87) | 80 (75-84) | 81 (77-86) | 85 (79-91) |
|  | Hours worked | 29 (25-32) | 28 (26-30) | 28 (24-31) | 28 (25-31) |
|  | Absenteeism | 5 (3-8) | 6 (4-8) | 7 (4-10) | 3 (1-5) |
|  | Presenteeism | 18 (13-23) | 16 (13-19) | 13 (9-17) | 11 (7-15) |
|  | Work impairment | 17 (12-21) | 14 (12-17) | 11 (8-14) | 10 (6-14) |
|  | Activity impairment | 20 (15-25) | 16 (14-19) | 14 (11-18) | 9 (6-12) |
|  | Wage loss | €657 (€288-€1157) | €668 (€402-€995) | €821 (€355-€1439) | €361 (€126-€679) |
| 6 months | Employment rate | 76 (67-86) | 83 (78-88) | 85 (80-90) | 84 (80-88) |
|  | Hours worked | 28 (23-34) | 28 (25-30) | 28 (25-31) | 30 (27-32) |
|  | Absenteeism | 4 (0-7) | 5 (3-7) | 3 (2-5) | 4 (2-6) |
|  | Presenteeism | 19 (9-29) | 15 (12-19) | 8 (5-12) | 9 (6-11) |
|  | Work impairment | 17 (9-24) | 14 (11-17) | 8 (5-10) | 8 (6-10) |
|  | Activity impairment | 21 (11-32) | 17 (14-21) | 11 (8-14) | 10 (8-12) |
|  | Wage loss | €420 (€28-€1080) | €611 (€318-€986) | €401 (€177-€693) | €509 (€257-€823) |
| 9 months | Employment rate | 80 (69-90) | 82 (77-87) | 82 (77-87) | 87 (83-91) |
|  | Hours worked | 26 (21-32) | 28 (25-32) | 29 (26-32) | 28 (26-30) |
|  | Absenteeism | 6 (0-13) | 2 (1-4) | 3 (1-5) | 4 (2-6) |
|  | Presenteeism | 9 (4-14) | 15 (11-19) | 12 (8-15) | 9 (7-12) |
|  | Work impairment | 7 (3-11) | 15 (11-19) | 11 (8-13) | 8 (6-10) |
|  | Activity impairment | 12 (4-21) | 19 (15-24) | 11 (8-14) | 11 (8-13) |
|  | Wage loss | €634 (€0-€1915) | €292 (€94-€560) | €361 (€127-€667) | €487 (€189-€853) |
| 12 months | Employment rate | 63 (37-88) | 80 (74-86) | 84 (81-88) | 87 (82-91) |
|  | Hours worked | 29 (20-38) | 30 (27-33) | 28 (25-31) | 27 (25-30) |
|  | Absenteeism | 7 (0-15) | 6 (3-9) | 3 (1-6) | 4 (2-6) |
|  | Presenteeism | 13 (2-24) | 14 (9-18) | 11 (7-14) | 8 (5-11) |
|  | Work impairment | 11 (3-19) | 13 (8-17) | 10 (7-13) | 7 (5-9) |
|  | Activity impairment | 18 (9-28) | 14 (10-18) | 12 (8-16) | 8 (6-11) |
|  | Wage loss | €619 (€0-€2519) | €718 (€266-€1321) | €422 (€126-€807) | €514 (€215-€892) |
| Responding parents’ employment rate, absenteeism, presenteeism, work impairment, and activity impairment at enrollment (Baseline), 3 months, 6 months, 9 months, and 12 months after diagnosis, by JIA activity. Estimates are adjusted means (95% CI) from GEE models with JIA activity, duration, age at diagnosis, and age group as covariates. Wage loss formula: (Absenteeism/100%) x 13 weeks x Work hours per week x Hourly wage x (Employment rate/100%) x 1 parent *R*^2^_marginal_ absenteeism = 0.12; *R*^2^_marginal_ presenteeism = 0.13; *R*^2^_marginal_ work impairment = 0.11; *R*^2^_marginal_ activity impairment = 0.13. | | | | | |

| **Table S3. Responding parents’ employment rate and hours worked by country** | | |
| --- | --- | --- |
| Country | Employment rate | Hours worked |
| Canada | 86% (35) | 31 (17) |
| Netherlands | 90% (30) | 24 (13) |
| Values are Mean (SD) | | |

Figure S1. Estimated wage loss due to absenteeism over time and by JIA activity state. Wage losses were estimated from 3 months prior to enrolment up to 12 months of participation.
